# Acupuncture therapy for tennis elbow: A protocol for systematic review and meta-analysis

**DOI:** 10.1097/MD.0000000000024402

**Published:** 2021-02-05

**Authors:** Yumei Zhou, Chen Chen, Yulin Yang, Haibo Yu, Zhuoxin Yang

**Affiliations:** The Fourth Clinical Medical College of Guangzhou University of Chinese Medicine, Shenzhen, GuangDong 518033, China.

**Keywords:** acupuncture, lateral epicondylitis, meta-analysis, protocol, systemic review

## Abstract

**Background:**

Acupuncture is widely used for analgesia in China and western countries. Lateral epicondylitis (LE) is a common disease, which influences the quality of life for many patients. The clinical practice indicates that acupuncture has a therapeutic effect on the LE; however, whether acupuncture is superior to sham acupuncture and some conventional therapy methods has been controversial. Hence, we will provide a protocol to explore the effectiveness and safety for acupuncture for LE.

**Methods:**

We will search only the randomized controlled trials (RCTs) literatures of acupuncture for LE from the following seven databases, including PubMed, Cochrane Central Register of Controlled Trials (The Cochrane Library), Excerpta Medica Database (EMBASE), China National Knowledge Infrastructure (CNKI), Chinese Biomedical Literature Database (CBM), Wanfang Data, and Chinese Scientific Journals Database (VIP). The Visual Analogue Scale (VAS) will be considered as the primary outcome and the secondary outcome will include effective rate and function recovery. Adverse events incidence caused by acupuncture will also be discussed, such as dizziness, nausea, vomiting, and weariness. The searching strategy, inclusion and exclusion criteria were made according to the principle of evidence-based medicine. The quality was evaluated by Cochrane Handbook for Systematic Reviews of Interventions (V5.1). All analyses will be conducted by Review Manager Software (V5.3).

**Result:**

The results of this review will be submitted to a recognized journal for publication.

**Conclusion:**

This proposed meta-analysis will assess the efficacy and safety of acupuncture therapy for LE.

## 1 Introduction

Lateral epicondylitis (LE), which is also named the tennis elbow, is a common orthopedic disorder with a prevalence of 1% to 3% in general population and 7% in handy workers.^[1]^ LE commonly involves in a collection of pain symptoms over the lateral epicondyle of the humerus, and motor function limitation, occurring most often in populations between 40 and 50 years with equal sex distribution.^[2]^ The underlying physiopathology of LE is not fully known, but it is considered to be caused by repeated overused of the wrist extensor muscles, particularly in extensor carpi radialis brevis that further activates the inflammatory processes.^[3]^ LE generally self-limiting last for 6 to 12 months^[4]^; however, persistent pain will be detected in the majority of patients even after 1 year. The pain and function limitation seriously influence on patients’ health and quality of life.

Nowadays, there are various treatments options for pain alleviation, such as steroid injections, nonsteroidal anti-inflammatory drugs (NSAIDs), and surgery, of which steroid injections and orally administered drugs are the most widely used treatment methods.^[5]^ Although these therapy methods have certain clinical curative effect, long-term use of them may induce increased risk of side effects, including weight gain, fatigue, sleep disturbance, and gastrointestinal intolerance. Because of the limitations associated with these conventional treatments, efforts have been made to identify effective, low-risk interventions.

As a common therapy and an important element of traditional Chinese medicine (TCM), the analgesic effect of acupuncture has been internationally recognized.^[6]^ For instance, a study conducted by Zhao^[7]^ found that acupuncture could exert a long-term reduction in migraine recurrence comparing acupuncture treatment with sham acupuncture and waiting-list groups. In addition, acupuncture was also found to lower the incidences of side effects compared with analgesics.^[8]^ Acupuncture is recommended for treatment of LE for many years, the efficacy and safety of this intervention has reported in many studies. However, whether acupuncture is superior to sham acupuncture or conventional treatments has not been systematically evaluated. A Cochrane review in 2002^[8]^ found that acupuncture could alleviate short-term pain of the lateral elbow, but the results were not pooled in meta-analysis because of the inclusion of only 2 studies. Another meta-analysis published in 2004^[9]^ explored the efficacy of acupuncture for LE compared with sham acupuncture, but no comparison with other conventional interventions, such as drugs and steroid injections, was conducted. Recently, more studies on this topic have been published, and the results remain conflicting. Therefore, this systematic review and meta-analysis of RCTs was performed to compare the efficacy of acupuncture with that of other therapies (sham acupuncture, drugs, and steroid injections) in LE.

## 2 Methods

### 2.1 Study registration

The protocol has been registered on the International Prospective Register of Systematic Reviews (registration number: CRD42019137973) basing on the Preferred Reporting Items for Systematic Reviews and Meta-Analyses Protocols (PRISMA-P) statement guidelines.

### 2.2 Ethics and dissemination

Ethical approval will not be required, as there will be no individual participants’ data in this systematic review. The results of this review will be disseminated by its publication of the manuscript in a peer-reviewed journal, aiming to provide information about effectiveness and safety for acupuncture for LE.

### 2.3 Eligibility criteria

#### 2.3.1 Research type

All available RCTs on acupuncture treatment for LE published from their inception to May 2019 will be included. Others such as retrospective study, case report will be excluded. There will be no restriction of languages.

#### 2.3.2 Participant type

Studies on patients who were diagnosed with LE (tennis elbow) will be included regardless of their age, gender, ethnicity, education, or economic status.

#### 2.3.3 Intervention measures

The purpose of this review is to provide information about the effectiveness and safety for acupuncture for LE. The intervention in the observation group limited acupuncture treatment (manual acupuncture and electro-acupuncture only), and controlled interventions will include sham acupuncture, blocking therapy, and western medicine.

#### 2.3.4 Outcome measures

The primary outcome measure is the changes of the Visual Analogue Scale (VAS). The secondary outcome will include effective rate and function recovery assessments. Adverse events incidence caused by acupuncture will also be discussed, such as dizziness, nausea, vomiting, and weariness.

#### 2.3.5 Exclusion criteria

The exclusion criteria are as follows: other types of studies, including non-RCTs, randomized crossover trials, retrospective studies, case reports, animal researches, and review studies, will be excluded; other participants, participants with severe physical or mental disease will be excluded; other types of interventions, the observation group of studies include other therapies except acupuncture, such as medicine, and the control group of studies contain any forms of acupuncture therapy; and full text unavailable.

### 2.4 Data sources

#### 2.4.1 Electronic searches

Some databases such as PubMed, Cochrane Central Register of Controlled Trials (The Cochrane Library), Excerpta Medica Database (EMBASE), China National Knowledge Infrastructure (CNKI), Chinese Biomedical Literature Database (CBM), Wanfang Data, and Chinese Scientific Journals Database (VIP) will be searched for eligible RCTs, which were published from inception to May 2019. The search strategy is summarized in Table 1 considering PubMed as an example, which was also suitable for other electronic databases.

**Table 1:** Search strategy of acupuncture for lateral epicondylitis in the PubMed database.

No.	Keywords
(1)	randomized controlled trial;
(2)	controlled clinical trials;
(3)	randomly;
(4)	randomized;
(5)	trial;
(6)	placebo;
(7)	1 or 2–6;
(8)	lateral humeral epicondylitis;
(9)	lateral epicondylitis;
(10)	tennis elbow;
(11)	8 or 9–10;
(12)	acupuncture;
(13)	acupuncture therapy;
(14)	acupoints;
(15)	body acupuncture;
(16)	scalp acupuncture;
(17)	electroacupuncture;
(18)	fire needle;
(19)	plum-blossom needle;
(20)	elongated needle;
(21)	intradermal needle;
(22)	12 or 13–21;
(23)	7 and 11 and 22

#### 2.4.2 Other searching resources

We will also filter relevant medical journals and magazines to find literature that is also eligible but not included in the above 7 databases. Relevant systematic review, meta-analysis, conference proceedings, or unpublished literature will be excluded. Moreover, we will filter relevant medical journals and magazines to identify literature, which is not included in the electronic databases.

### 2.5 Study selection and data extraction

#### 2.5.1 Study selection

**Figure 1:**
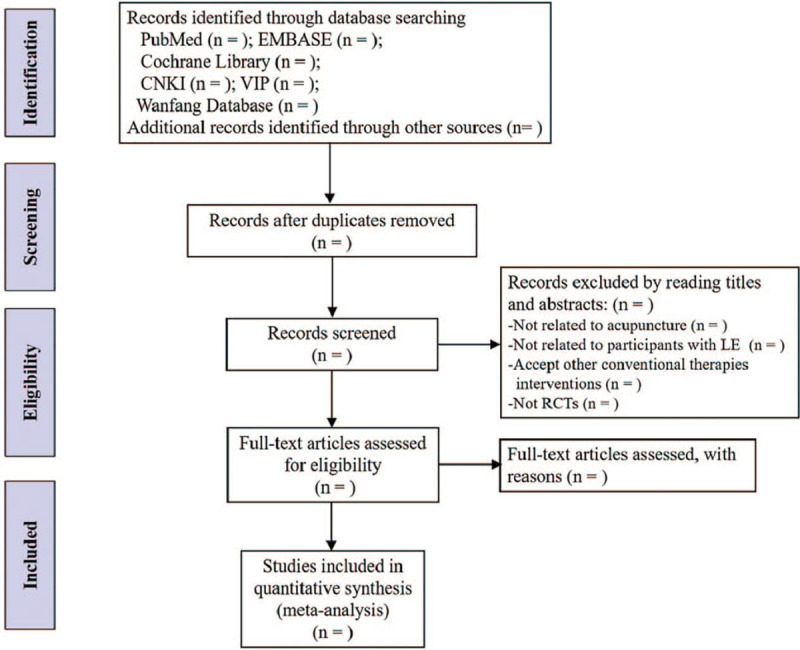
Flow diagram of study selection process.

Two authors (ZYM and CC) will screen the titles and abstracts to filter the eligible trials according to the inclusion criteria independently. Then, full text will be read if needed. Excluded studies will be listed in a table with the reasons for their exclusion. Disagreements will be solved by discussing with a third reviewer. The flow chart of study selection procedure according to Preferred Reporting Items for Systematic Reviews and Meta-Analyses (PRISMA)^[10]^ is shown in Figure 1.

#### 2.5.2 Data extraction and management

Two authors will extract the data independently, and discuss the discrepancies to reach an agreement. Data including basic information of the included finding (publication year, author, etc); essential information of the included participants (e.g., number of participants, gender, age, etc); interventions details; randomization methods used (registry platform, sample size, blinding method); and outcome, measures (VAS score and effective rate). Missing data will be obtained by contacting trail author for unreported information.

### 2.6 Data analysis

#### 2.6.1 Assessment of risk of bias in included studies

According to the Cochrane Handbook, the risk of bias of each included RCT will be evaluated in the following seven consequences: random sequence generation, allocation concealment, blinding of participants and personnel, blinding of outcome assessments, incomplete outcome data, selective reporting, and other bias. All the included studies will be classified into 3 categories: “low risk of bias,” “high risk of bias,” and “unclear risk of bias.” In addition, the overall evidence levels of the primary and secondary outcomes of acupuncture for LE were assessed using the Grading of Recommendations, Assessment, Development, and Evaluation (GRADE) system. Two reviewers will independently perform evaluations of the methodological quality of each included study. Any disagreements or doubts were figured out by discussion or by consulting another author.

#### 2.6.2 Statistical analysis

Meta-analysis and test sequential analysis (TSA) of the included studies will be handled respectively by Review Manager Software (V5.3). As to evaluating the continuous variables, we choose mean difference (MD) and 95% confidence intervals (95% CIs). When it comes to dichotomous variables, we adapt rate ratio and 95% CIs, which were used to evaluate the extracted data.

#### 2.6.3 Addressing missing data

We will contact trial authors for missing or unreported data if necessary. If failed, we will discuss about the impact of the missing data and decide whether to omit it from the data synthesis or using a limited analysis to perform it.

#### 2.6.4 Data synthesis and subgroup analysis

We will use Review Manager Software (V5.3) to conduct a meta-analysis if it is possible. We will combine the relative risks for dichotomous outcomes and MDs for continuous outcomes both with 95% CIs. If heterogeneity is significant (*I*^2^ ≥ 50%), we will apply a random-effects model; otherwise (*I*^2^ > 50%), we will use fixed-effect model. If heterogeneity is significant (*I*^2^ ≥ 50%), a subgroup analysis will be performed based on different types of acupuncture (body acupuncture, intradermal acupuncture, electro-acupuncture, fire needling, warm acupuncture, elongated needle therapy, laser acupuncture, etc).

#### 2.6.5 Assessment of heterogeneity

We will search for overlapping CIs in forest plots and use a χ^2^ test for statistical heterogeneity and the *I*^2^ statistic (*I*^2^ > 50% shows the existence of heterogeneity) to estimate the level of heterogeneity across the studies.

#### 2.6.6 Assessment of publication bias

We will use funnel plots to figure out the reporting bias of the studies. If more than 10 studies are included in the meta-analysis, we will use Egger method^[11]^ to explain the asymmetry. If the points are symmetrically distributed on both sides, we will consider that the result is reliable, but if it allocated asymmetrically, we will consider the reporting bias exists and the result is less reliable.

#### 2.6.7 Sensitivity analysis

If the heterogeneity is high, we will conduct a sensitivity analysis to verify quality and robustness of the meta-analysis result on a basis of sample size, methodological quality, and the effect of missing data.

#### 2.6.8 Grading the quality of evidence

We will classify the quality of evidence of the included studies based on guidelines of the GRADE (Grading of Recommendations, Assessment, Development, and Evaluation).^[12]^ The evidence quality will be ranked by 4 levels: high, moderate, low, or very low.

## 3 Discussion

A systematic review has shown the effectiveness and safety of acupuncture for the treatment of LE, but the evidence of efficacy and safety of acupuncture comparing other conventional interventions is insufficient, so that clinicians cannot judge the advantage of acupuncture, which will be not conducive to the popularization and application of acupuncture for LE. Therefore, it is imperative to conduct a systematic review and meta-analysis of available literature to evaluate the clinical efficacy and safety of acupuncture on LE objectively, in order to provide reliable evidence-based medical evidence for clinical promotion and efficacy evaluation of acupuncture treatments for LE. The protocol has been registered in the platform of international prospective register of systematic reviews (PROSPERO), and it will be conducted strictly according to steps of systematic review. In addition, the quality of evidence for main outcomes will be assessed with the GRADE approach. The process of performing this systematic review, shown in Figure 1, will include 4 parts: identification, study inclusion, data extraction, and data synthesis. Collection of data is continuing for this study. In this review, we will also provide clinical outcome measures, therapeutic effect, adverse reactions, and side effects of acupuncture with objective statistics and rigorous analysis. Therefore, we hope that this meta-analysis will confirm the efficacy and safety of acupuncture on LE, and provide more evidence sources for acupuncture in the treatment of LE. What is more, results will offer reliable references for clinicians and patients in clinical decision-making.

## Author contributions

Yumei Zhou designed the study. Yumei Zhou and Chen Chen drafted the protocol. All authors revised the manuscript. All authors approved the final version.

**Conceptualization:** Yumei Zhou.

**Data curation:** Chen Chen.

**Formal analysis:** Yulin Yang.

**Funding acquisition:** Zhuoxin Yang.

**Methodology:** Haibo Yu.

**Project administration:** Yumei Zhou, Zhuoxin Yang.

**Validation:** Haibo Yu.

**Writing – original draft:** Yumei Zhou, Chen Chen.

**Writing – review & editing:** Yumei Zhou.
